# A Feasibility Cluster Randomized Controlled Trial of Individual Placement and Support (IPS) for Patients With Offending Histories

**DOI:** 10.3389/fpsyt.2019.00952

**Published:** 2020-01-13

**Authors:** Najat Khalifa, Emily Talbot, Shaun Barber, Justine Schneider, Yvonne Bird, Julie Attfield, Peter Bates, Dawn-Marie Walker, Birgit Völlm

**Affiliations:** ^1^ Department of Psychiatry, Queen’s University, Kingston, ON, Canada; ^2^ Wells Road Centre, Nottinghamshire Healthcare NHS Foundation Trust, Nottingham, United Kingdom; ^3^ DOCS contracted partner of Amgen, Cambridge, United Kingdom; ^4^ Clinical Trials Unit, University of Leicester, Leicester, United Kingdom; ^5^ School of Sociology and Social Policy, University of Nottingham, Nottingham, United Kingdom; ^6^ Corporate Services, Nottinghamshire Healthcare NHS Foundation Trust, Nottingham, United Kingdom; ^7^ Public and Patient Involvement, The Institute of Mental Health, Nottingham, United Kingdom; ^8^ Health Sciences, University of Southampton, Southampton, United Kingdom; ^9^ Department of Forensic Psychiatry, Universitätsmedizin Rostock, Rostock, Germany

**Keywords:** individual placement and support, feasibility, employment, offenders, mental disorder

## Abstract

**Objective:** To examine the feasibility of conducting a fully powered randomized controlled trial (RCT) of Individual Placement and Support (IPS). IPS is a form of supported employment which aims to put people into open employment quickly and in accordance with their preferences. It is delivered by employment specialists collocated within clinical teams, and provides time unlimited support for the individual and their employer, along with welfare benefits counselling.

**Method:** A feasibility cluster RCT of treatment as usual (TAU) plus IPS versus TAU alone was conducted over 12 months among patients with offending histories in a community forensic setting in the UK. The feasibility criteria were to achieve 50% recruitment rate; 50% completion rate for IPS; 50% completion rate of all outcome measures; and 80% acceptability rating for IPS. The primary efficacy outcome was the proportion of people in open employment at 12 months. The secondary outcomes were other vocational and educational activities; Brief Psychiatric Rating Scale; Rosenberg’s Self-esteem Scale; Client Service Receipt Inventory; quality of life using the SF12-v2 and EQ5-D3; Social Functioning Questionnaire; Work Limitation Questionnaire; and reoffending.

**Results:** Participants’ mean age was 39.2 years. The majority were male (88.9), White British (72.2), and single (72.2%). Over 72% had no higher qualification beyond secondary education; mean years in education was 10.4. Over one third had schizophrenia, one fifth had depression, and the rest had personality disorder as their primary diagnosis. Participants had a lifetime average of 7.5 convictions for 15.5 offences. The recruitment rate of all referrals was 38.3% (IPS n = 11; TAU n = 7). Completion rate for IPS was 54.5, with 45.5% acceptability rating. Completion rates for outcome measures for the groups at baseline and 12 months ranged from 22.2 to 100%. The proportion of people in open employment at 12 months were 9.1 and 0% for IPS and TAU respectively.

**Conclusion:** It is not feasible to conduct a full RCT of IPS in community forensic settings in the UK owing to recruitment and retention difficulties. Conducting a trial of this kind requires a large pool of patients from multiple sites and longer IPS implementation and recruitment periods than those of this study.

**Clinical Trial Registration:** www.ClinicalTrials.gov, identifier NCT02442193.

## Introduction

### Mental Disorders Among Offenders

In the UK, 1 in 5 people who are of working age have mental health problems (1). Mental disorders are particularly prevalent among those who are in conflict with the law in correctional (2) and community psychiatric settings (3), and those on probation (4) with higher rates being reported for younger individuals (5). The Office for National Statistics survey of psychiatric morbidity among prisoners in England and Wales (2) reported high prevalence rates for personality disorder (78 for male remand, 64 for male sentenced, 50% for female prisoners), neurotic disorders (59 for male remand, 40 for male sentenced, 76% for female remand), and functional psychosis such as schizophrenia and manic depression (10 for male remand, 7 for male sentenced, 14% for female prisoners). Similarly, analysis of data for young offenders aged 16 to 20 years (6) recorded high prevalence rates for personality disorder (84 for male remand, 88% for male sentenced), and functional psychosis (8 for male remand, 10 for male sentenced, 9% for female sentenced).

### Employment Support

In the UK, significant proportions of offenders with mental disorders are unemployed (7). Niven and Stewart (8) reported that in 2003, only 30% of offenders released from prison achieved positive employment, training, or education outcomes. A more recent survey in 2012 (9) reported significantly higher rates of unemployment among people on probation (60.7) than in the general population (7.9%). Similarly, unemployment is highly prevalent among people discharged from forensic mental health services in the UK. These services provide psychiatric treatment for individuals with both mental disorders and offending histories (henceforth referred to as patients with offending histories) in secure forensic hospital and community settings. There are poor long-term employment outcomes for this group of people. Davies and colleagues (10) reported on the long-term outcomes of 550 patients discharged from a medium secure unit in England over a 20-year period. They reported that only 14.5% were in competitive employment which was mostly provided by relatives. Using data from the same study, Sahota et al. (11) reported that only 13.5% of women secured employment over the same follow-up period.

This attributes to offenders with mental disorder faring less well than their non-offender counterparts on measures of social problem-solving skills, socio-economic deprivation, self-esteem, quality of life, and mortality (12–16). This is not surprising since employment has been linked to several desirable outcomes including income, social integration, enhanced self-esteem, a sense of optimism (17–19), and reduction in re-offending rates (20, 21).

Therefore, existing literature and government initiatives emphasized the importance of using work as a means to improve health outcomes (17–19), and reduce re-offending rates among offenders (22). However, barriers to employment among patients with offending histories are numerous, including stigma, homelessness, substance misuse, negative attitudes among employers, and lack of relevant skills and qualifications (7, 23, 24). Furthermore, evidence from the UK suggests that while it is possible to support offenders with mental disorders into mainstream employment, only a minority of these individuals are offered help (7). For instance, a recent review of the literature on employment of ex-prisoners with severe mental illness documented a specific lack of employment opportunities for these individuals (25) who encounter a myriad of barriers to employment including stigma, social isolation, substance misuse, and low educational attainment (26). Furthermore, Talbot and colleagues (27) reviewed the evidence base for work skills program for offenders with mental disorders, and reported that while a range of employment program have been developed for these individuals, the evidence base for their effectiveness is limited in terms of impact on mental health, substance misuse, or reoffending rates.

There is a dearth of studies on the provision of employment support for patients with offending histories in the UK. We identified three studies that specifically reported on outcomes from programs that provided employment support for these individuals. Garner (28) described a prevocational training program that provided employment support within a medium secure unit in England. This program facilitated patient access to training that adjusted for the unique needs of this population, in terms of fluctuating mental health, medication, lack of knowledge about vocational activities, pace of learning, and being subject to legal jurisdictions. McSweeney and Hough (29) reported on outcomes from a five-year government sponsored scheme in London, “From Dependency to Work”, that supported offenders with multiple needs including mental health, substance use, and literacy problems. They reported that the success of the scheme was limited due difficulties in effectively identifying those with multiple needs and planning interventions as well as organizational challenges. More recently, Samele, Forrester, and Bertram (30) evaluated an Employment and Social Inclusion Project which was developed and piloted to support patients with forensic histories into employment and vocational activities. They reported that of the 57 individuals who engaged with the project, only 4 (7.0) gained competitive employment, and 8 (14.0%) gained other paid employment.

### IPS

There are some indications that Individual Placement and Support (IPS) can potentially help secure competitive employment for patients with offending histories. Although the current literature supports the effectiveness of IPS in general psychiatric settings (31), the evidence base for its effectiveness in forensic mental health services is limited. These services provide psychiatric treatment for patients with offending histories and those who pose significant risks to others because of their mental disorder (32). These services provide a range of interventions including risk assessment and case management, and some provide specific psychotherapeutic interventions for people with personality disorder, sex offenders, or those with substance use disorders (33). A study in the USA that assessed the effectiveness of IPS versus a job club approach with peer support for people with severe mental illness and justice involvement reported that IPS was superior to the control intervention (34). In the UK, Durcan et al. (35) reported on the effectiveness of IPS for those leaving prison with mental health disorders. In total, the project supported 21 people into competitive employment (39% of those meeting the project inclusion criteria). However, this study did not employ a randomized controlled trial design, and the use of IPS was limited by lack of integration into local mental health services. Beck and Wernham (36) described outcomes from several business enterprises, underpinned by the principles of IPS, across two forensic mental health units in East London. They reported that these enterprises provided patients with the essential skills required to secure gainful employment upon discharge including punctuality, customer service, self-presentation, and employer references. However, the authors did not report quantitative data to support their assertions.

The present study was needed to pave the way for robust randomized controlled trials (RCTs) of IPS for patients with offending histories in the community, so that this intervention, proven to be effective in adults with mental health problems could be appraised for its potential to these individuals to live more rewarding lives, reduce re-offending, and minimize their reliance on statutory services.

IPS is regarded as a complex intervention since it involves several interacting components. Developing an evidence base for IPS in a forensic mental health setting adds to this complexity, since the management of patients with offending histories combines various treatment modalities to address mental health issues, offending behavior, and risk management (37). It is the same complexities in the practice of forensic mental health that make the implementation and evaluation of IPS a challenge.

The challenges associated with IPS implementation in this study are described in detail elsewhere (38, 39). In short, barriers to IPS implementation were numerous including competing interests between IPS and psychological therapies, staff perceptions about patients’ readiness for work, negative staff attitudes towards IPS, difficulty engaging employers, lack of employment related performance indicators in health services, and concerns about the impact of returning to work on welfare benefits. Employers regarded offending history as a key barrier to employing patients with offending histories. Facilitators of IPS implementation included communicating the benefits of IPS to stakeholders, support from healthcare managers, and interdisciplinary collaboration. Our findings highlighted the challenges associated with implementation of IPS in forensic mental health settings, which requires robust planning and collaboration with internal and external agencies.

Due to the challenges associate with IPS implementation and the financial implications of conducting a fully powered RCT of IPS among patients with offending histories, a feasibility study was necessary to determine the parameters required to conduct a full trial, in terms of sample size, recruitment rates, and completion rates for both the intervention and outcome measures. This is particularly important in intervention trials that involve a blending of several interacting components such as IPS (40).

## Present Study

The primary aim of this study was to examine the feasibility of conducting a full RCT to evaluate the effectiveness of IPS in improving employment and psychosocial outcomes for forensic psychiatric populations in the community. The specific objectives of the study were to:

- assess the feasibility of conducting a full trial according to predetermined criteria;
- estimate the parameters required to conduct a full RCT in terms of sample size, recruitment rates, and completion rates for both the intervention and outcome measures; and
- estimate the means and ranges of questionnaire data and pattern of missing data.

Based on the recruitment rate in another IPS trial in general community mental health settings in the same city (41), and feasibility criteria set out by another trial in the same service in which the feasibility study was conducted (42), we proposed that a definitive trial would be considered feasible if:

1. The recruitment rate to the project was at least 50% of all referrals.
2. Fifty percent completion rate for those assigned to the intervention was achieved.
3. Eighty percent of those assigned to IPS would find the intervention acceptable (a score of more than 3 on a 5-point Likert scale indicated acceptability).
4. Fifty percent of participants had completed all outcome measures at baseline and follow-up.

## Method and Materials

### Design

The Individual Placement and Support for patients with offending histories (IPSOH) trial (43) entailed conducting a feasibility cluster randomized controlled trial over 12 months involving four clusters. These were defined according to the clinical configuration of a county wide community forensic service in Nottinghamshire, England, which included four major divisions:

Cluster 1: City Community Forensic Service.
Cluster 2: County Community Forensic Service.
Cluster 3: City Personality Disorder Service.
Cluster 4: County Personality Disorder Service.

### Sample and Settings

Individuals aged 18 years or over who were on the caseloads of the community forensic services were eligible to participate in the study. Patients who were unable to provide informed consent, not eligible to work in the UK, currently in open employment, or did not wish to work were not invited to participate.

The Nottinghamshire community forensic service provides treatment for patients with offending histories across four major divisions; two mainstream community forensic and two personality disorder services. The community forensic services provide case management services for people with major mental disorder (e.g., schizophrenia, mood disorders, personality disorder) or intellectual disability who are in conflict with the law or those who pose significant risks to others as a result of their mental disorder or intellectual disability. The personality disorder services are therapy only services which provide psychotherapeutic interventions, such as psychodynamic psychotherapy, social problem solving, and dialectal behavioral therapy, for people with personality disorders including those with or without offending histories. At the start of the study, almost 80% of the 250 patients who were on the caseloads of the Nottinghamshire Community forensic service were unemployed, indicating that the IPS service could potentially enable these individuals to fulfil their employment aspirations as a part of their recovery.

### Randomization and Blinding

Randomization was carried out by an independent statistician who allocated clusters 1 and 4 to the intervention arm [treatment as usual (TAU) plus IPS], and the other two clusters to the control arm (TAU alone). IPS was provided by an employment specialist who worked across the two clusters assigned to IPS. Patients in all clusters continued to receive treatment as usual from the Nottinghamshire community forensic service. This was an open label study. Participants, clinicians, and researchers were aware of the intervention allocation.

### Interventions

The interventions comprised TAU+IPS versus TAU alone. Individuals assigned to the intervention arm received ongoing support from the employment specialist in accordance with IPS principles. The employment specialist worked closely with those assigned to the intervention arm. This entailed beginning job searches rapidly based on individual preferences; providing individualised support to both the patient and their employer; and providing welfare benefits counselling to support the transition from benefits to work. Co-location of the employment specialist within clinical teams allowed information about risks to be shared between employers, health and other agencies, and subsequently taken into consideration when matching jobs to individual preferences.

TAU comprised clinical case management within mainstream community forensic services or psychotherapy only within personality disorder services. Clinical variations in TAU were taken into consideration as part of the randomization procedure such that each study arm comprised of one mainstream community forensic service and one personality disorder service.

### IPS Implementation and Fidelity Reviews

Details of IPS implementation and fidelity reviews are reported elsewhere (38, 39). In brief, due to funding constraints, the IPS service model was implemented prior to the start of the feasibility study over a relatively short period, only 6 months, in accordance with the Consolidated Framework for Implementation Research (CFIR) (44). The CFIR consists of five constructs: characteristics of the intervention, inner setting, outer setting, individuals involved, and implementation process. An employment specialist supervised by a senior occupational therapist, delivered IPS, and an IPS steering group was established to oversee the IPS implementation and delivery. IPS fidelity reviews were conducted using the UK version of the IPS fidelity scale (45) by an independent IPS expert at the start and end of the implementation period to assess how closely the IPS service adhered to the principles of IPS. The fidelity scale is scored out of 125 with higher scores denoting greater degrees of implementation: 115–125 = exemplary fidelity; 100–114 = good fidelity; 74–99 = fair Fidelity; 73 and below = not supported employment. A total fidelity score of 61 at baseline, reflected lack of employment support in the community forensic services. In contrast, at end of the implementation period, a fair degree of fidelity (total IPS fidelity score = 85) was achieved across the two IPS clusters. No further fidelity reviews were conducted due to funding constraints.

### Assessments

Assessments of participants took place in community forensic team sites at baseline, 6 months and 12 months, using a data collection tool and several scales as follows.

#### Baseline

1. Information concerning socio-demographics, diagnosis, and offending history was collected using a data collection tool designed for this study. Socio-demographic data included age, gender, ethnicity, number of years in education, and qualifications. Information on diagnosis was obtained from their current psychiatrist. Offending history was determined from case files and Police National Computer records.
2. Brief Psychiatric Rating Scale (BPRS) (46): This is an 18-item clinician/researcher rated scale used to measure psychiatric symptoms such as somatization, anxiety, depression, hallucinations, and others. Each item is measured on a scale of 1 to 7 (1 = not present, 2 = very mild, 3 = mild, 4 = moderate, 5 = moderately severe, 6 = severe, 7 = extremely severe).
3. Social Functioning Questionnaire (SFQ) (47): This is a clinician/researcher rated scale used to assess social functioning. It is divided into 5 sections, each containing 8 items: Self-care Skills, Domestic Skills, Community Skills, Social Skills, and Responsibility. Of these, ten items are marked as “Index Items” which can be used to derive a global measure of social functioning.
4. Rosenberg’s Self-Esteem Scale (48): This is a self-rated scale which measures self-esteem on ten items. Each item is measured on a 4-point Likert scale— from strongly agree to strongly disagree.
5. Work Limitations Questionnaire (WLQ) (49): This is a self-rated questionnaire that measures the degree with which health problems impact on specific aspects of job performance and the productivity impact of these limitations. Respondents are asked to rate their performance on 25 specific job demands, yielding four work limitation demands: Time Management, Physical demand, Mental/Interpersonal demands, and Output demands.
6. Health-related quality of life: This was assessed using the European Quality of Life Scale EQ5-D (50) and Short Form 12 item Health Survey – version 2 - SF-12v2 (51). EQ5-D is a self-rated measure of health status that provides a measure of health for clinical and economic appraisal. It provides a descriptive profile and single index value for health status that can be used in economic evaluations of health care. SF-12v2 is a 12 item self-rated questionnaire survey that measures functional health and well-being from the patient’s perspective.
7. Client Service Receipt Inventory (CSRI) (52): This scale is used to capture data on recent use of health and social care services, accommodation and living situation, income, employment, and benefits. Data collected using the CSRI were used to calculate the costs of health and social care using the 2016-unit costs of health and social care (53) and the National Health Service (NHS) reference costs 2015-2016 (54).

#### Follow Up Data

At 6 months, information about employment activities (e.g., job tenure, hours in paid work, type of work, and income) was collected by asking participants structured questions about these activities. Data on educational activities were also collected as these may be a more feasible outcome for younger patients. Data on other vocational activities such as training and volunteering were collected. Additionally, the other outcome measures including BPRS, SFQ, Rosenberg’s Self-Esteem Scale, WLQ, SF-12v2, EQ5-D, and CSRI were repeated.

At 12 months, information about employment and educational activities was collected and all the other outcome measurers repeated as above. Additionally, re-offending data for the 12 months following randomization was obtained.

### Primary and Secondary Efficacy Outcomes

The primary efficacy outcome was the proportion of people in open employment at 12-month follow-up. Open employment was defined as having a job paying at least the minimum wage in a mainstream setting and not specifically for people with disability or special needs. This was in accord with outcome measures used in another IPS trials in the UK (55).

The secondary efficacy outcomes included other employment and educational activities, questionnaire outcomes (BPRS, SFQ, Rosenberg’s Self Esteem Scale, WLQ, Health-related quality of life using SF-12v2 and EQ5-D, CSRI) and re-offending rates.

### Sample Size

The sample size was calculated based on the recommendations of Eldridge and Kerry (56). This yielded a total sample size of 76 across four clusters (38 per study arm). According to Eldridge and Kerry (56), for samples of 75–150 individuals, 95% CI for intra-class correlation coefficient (ICC) estimate is similar whether four or eight clusters are used.

### Data Analysis

The analytic strategy was initially tailored to meet specific objectives of the feasibility study, for the whole sample and across the clusters. However, due to the small sample size, it was not possible to present results by cluster. Analysis was conducted on an intention to treat basis. Descriptive statistics were used to describe recruitment and retention rates, medians and ranges of efficacy outcome measures (both primary and secondary) and patterns of missing data.

### Research Ethics

The study received research ethics approval from the East Midlands-Nottingham 1 Research Ethics Committee (Ref. 15/EM/0253). Written informed consent was obtained from all participants.

## Results

### Sample Characteristics

**Figure 1:**
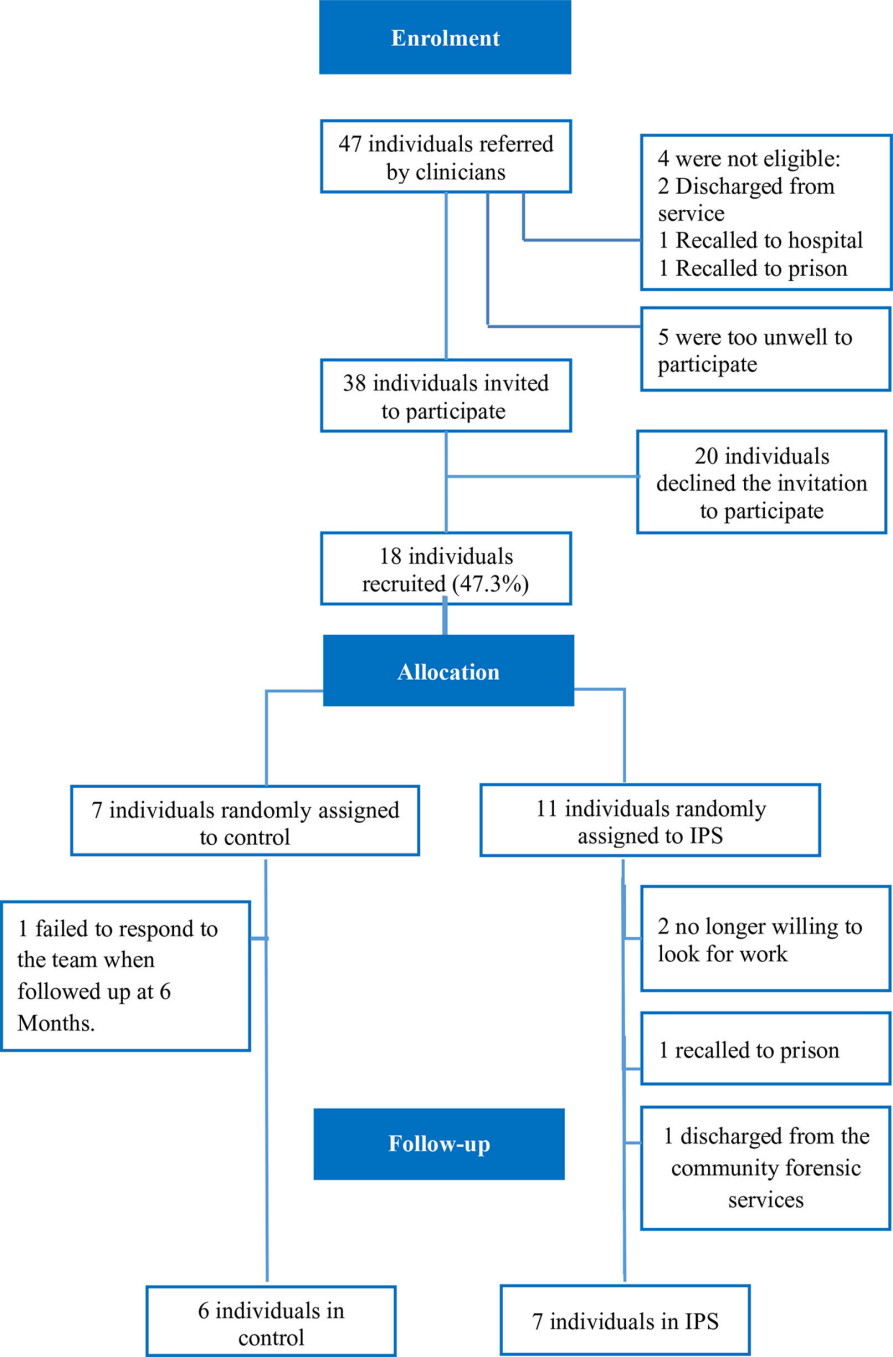
CONSORT flow chart.

The CONSORT flow chart (**Figure 1**) summarizes the recruitment pathway. Clinicians referred 47 patients. Of these, four patients were not eligible, and five were too unwell to participate. Of the remaining 38, 18 were recruited (38.3% recruitment rate) – 7 to the control arm and 11 to the IPS arm. Later, one control and four IPS participants dropped out.

Participants’ mean age was 39.2 (range = 24-53). The majority were male (88.9), White British (72.2), and single (72.2%). Over 72% had no higher qualification beyond secondary education; mean years in education was 10.4 (range = 2-13). Over one third had schizophrenia, one fifth had depression, and the rest had a personality disorder as their primary diagnosis. Participants had a lifetime average of 7.5 (range = 1-20) convictions for 15.5 (range = 1-50) offences. See also **Table 1** for more information.

**Table 1:** Sample characteristics at baseline.

	IPS	Controls	Overall
Mean age (range)	37.2 (24, 51)	42.3 (25, 53)	39.2 (24, 53)
Gender Male, n (%)	9 (81.8)	7 (100)	16 (88.9)
Ethnicity, n (%)			
White British	7 (63.6)	6 (85.7)	13 (72.2)
Black	3 (27.3)	0 (0.0)	3 (16.7)
Mixed	1 (9.1)	1 (14.3)	2 (11.1)
Marital status, n (%)			
Single/unmarried	8 (72.7)	5 (71.4)	11 (72.2)
Married	2 (18.2)	0 (0.0)	2 (11.1)
Separated	0 (0.0)	1 (14.3)	1 (5.6)
Divorced	1 (9.1)	1 (14.3)	2 (11.1)
Highest qualification, n (%)			
Primary education or less	2 (18.2)	1 (14.3)	3 (16.7)
Secondary education:	5 (45.5)	5 (71.4)	10 (55.6)
Tertiary/further education	4 (36.4)	1 (14.3)	5 (27.8)
Mean years of education (range)	10.8 (9, 13)	9.9 (2, 12)	10.4 (2, 13)
Diagnosis, n (%)			
Schizophrenia	4 (36.4)	2 (28.6)	6 (33.3)
Major depression	2 (19.1)	2 (28.6)	4 (22.2)
Personality disorder	5 (45.5)	3 (42.8)	8 (44.5)
Mean number of convictions across life time (range)	8.6 (1, 20)	4.7 (2, 9)	7.5 (1, 20)
Mean number of offenses across life time (range)	19.2 (1, 50)	6.3 (2, 9)	15.5 (1, 50)
Mean number of offenses against the person (range)	4.3 (0, 13)	2 (0, 6)	3.7 (0, 13)
Mean number of sexual offenses (range)	0.1 (0, 1)	0 (0.0)	0.1 (0, 1)
Mean number of offenses against property (range)	2.8 (0, 13)	2.5 (0, 6)	2.7 (0, 13)
Mean number of theft and kindred offenses (range)	4.6 (0, 17)	1 (0, 3)	3.7 (0, 17)
Mean number of fraud and kindred offenses (range)	0.1 (0, 1)	0 (0)	0.1 (0, 1)
Mean number of other offenses (range)	1.5 (0, 8)	0.7 (0, 2)	1.3 (0, 8)
Mean number of drug offenses (range)	0.5 (0, 2)	2.3 (0, 9)	2.0 (0, 9)
Mean number of firearm/shotgun/offensive weapon offenses (range)	0.5 (0, 5)	0 (0)	0.5 (0, 5)
Mean number of public order offenses (range)	1.9 (0, 7)	0.3 (0, 1)	1.5 (0, 7)
Mean number of vehicle/driving offenses (range)	0.3 (0, 2)	0 (0)	0.2 (0, 2)

### IPS Fidelity

There was no formal provision for IPS prior to the start of the project. A fair degree of IPS fidelity was achieved at the end of the implementation period (total fidelity score = 85).

### Feasibility Outcomes

Recruitment rates were 38.3 of eligible referrals (18/38) and 47.4% of all referrals (18/47). Completion rate for IPS was 54.5 (6/11), with 45.5% acceptability rating (see **Table 2**).

**Table 2:** Feasibility outcomes.

Feasibility outcome	Success criteria	Observed
Recruitment rate	≥50% of all referrals	38.3% (18/47) of all referrals [47.4% (18/38) of eligible referrals]
Completion rate of intervention	≥50%	54.6% (6/11)
Acceptability rate of intervention^a^	≥80%	45.5% (5/11)^b^
Complete outcome measurements at baseline & 12 months	≥50%	5.6% (1/18) [Intervention: 0.0% (0/11) Control: 14.3% (1/7)]

a Acceptable (a score of more than 3 on a 5-point Likert scale indicates acceptability [at 12 month follow-up].

b All five participants that answered this question gave a Likert of at least 3.

Data on the primary efficacy outcome was available for all but one participant (17/18; 94.4%) at 12 months. Respective completion rates for secondary outcomes for the groups at baseline and 12 months were 17/18 (94.4) v. 4/18 (22.2%) for BPRS; and 18/18 (100) v. 8/18 (44.4%) for SFQ, Rosenberg’s, SF12-v2, EQ-5D, and CSRI. Completion rates for outcomes at 7–12 months for the groups were 15/18 (83.3%) for reoffending data; 10/18 (55.6%) for h worked/week; 8/18 (44.4%) for days employed and h in education/week; 16.7% for WLQ Productivity Loss, and 9/18 (50%) for change in qualification. Full data was available for only one participant (5.6%).

### Efficacy Outcomes Measures

#### Primary Efficacy Outcome

The proportions of people in open employment at 12 months were 9.1 (1/11) and 0% for the IPS and control groups respectively.

#### Other Employment and Education Outcomes

Average hours worked per week (IPS v. controls) were: 0.8 (0–4.6) v. 0 (0) [1^st^ 6 months] and 0.6 (0–3.8) v. 0(0) [2^nd^ 6 months]. Mean number of days employed were 12.2 (0–73) v. 4.4 (0–22) [1^st^ 6 months] and 44.8 (0–179) v. 3.25 (0–3) [2^nd^ 6 months]. Number of days was counted in calendar days regardless of h worked per week and included charity work. Figures for controls represent voluntary work for a third sector charitable organization. Over 14% (IPS) of participants attained a higher qualification during the study period.

#### Questionnaire Data

**Table 3** summarizes questionnaire data in terms of means, ranges, and patterns of missing data.

**Table 3:** Questionnaire data at baseline, 6 months, 12 months and change from baseline at 12 months.

Items	IPS N = 11				Controls N = 7			
	Baseline Mean (range) N Missing	6 months Mean (range) N Missing	12 months Mean (range) N Missing	Change from baseline at12 months Mean (range) N Missing	Baseline Mean (range) N Missing	6 months Mean (range) N Missing	12 months Mean (range) N Missing	Change from baseline at12 months Mean (range) N Missing
BPRS scores	29 (21, 26) 10 1	31 (24, 45) 6 5	34 (26, 42) 2 9	-5.0 (-4, -6) 2 9	34.4 (26, 41) 7 0	27.3 (19, 37) 6 1	25.5 (19, 32) 2 5	-7.5 (-7, -8) 2 5
Rosenberg’s self-esteem scores	15.9 (22, 5) 11 0	17.2 (24, 9) 6 5	17 (26, 8) 4 7	4.5 (-1, 15) 4 7	16.3 (11, 20) 7 0	16.3 (11, 22) 6 1	17.5 (12, 20) 4 3	-7.5 (-7, -8) 4 3
WLQ scores								
Time	N/A 0 11	33.3 (10, 60) 3 8	33.3 (0, 85) 3 8	N/A 0 11	N/A 0 7	66.3 (62.5, 70) 2 5	N/A 0 7	N/A 0 7
Physical	N/A 0 11	16.7 (0, 37.5) 3 8	27.8 (0, 41.7) 3 8	N/A 0 11	N/A 0 7	85.6 (81.3, 90) 2 5	N/A 0 7	N/A 0 7
Mental	N/A 0 11	28.7 (13.9, 58.3) 3 8	37 (5.5, 75) 3 8	N/A 0 11	N/A 0 7	36.1 925, 47.2) 2 5	N/A 0 7	N/A 0 7
Output	N/A	32.5 (0, 65) 2 9	33.6 (5, 62.5) 3 8	N/A 0 11	N/A 0 7	42.7 (16.7, 68.8) 2 5	N/A 0 7	N/A 0 7
WLQ Index	N/A 0 11	0.09 (0.02, 017) 2 9	0.10 (0.01, 0.19) 2 9	N/A 0 11	N/A 0 7	0.14 (010, 0.18) 2 5	N/A 0 7	N/A 0 7
WLQ Productivity Loss	N/A 0 11	8.8 (2.2, 15.4) 2 9	9 (1.1, 17.6) 2 9	N/A 0 11	N/A 0 7	13.2 (9.9, 16.5) 2 5	N/A 0 7	N/A 0 7
SF12-v2 scores								
Physical function	75 (25, 100) 11 0	75 (25, 100) 6 5	87.5 (75, 100) 4 7	6.3 (0, 25) 4 7	60.7 (0. 100) 7 0	83.3 (25, 100) 6 1	75 (0, 100) 4 3	7.3 (0, 25) 4 3
Role Physical	63.8 (25, 100) 10 1	91.7 975, 100) 6 5	84.4 (50, 100 4 7	8.3 (0, 12.5) 4 7	58.9 912.5, 100) 7 0	60.4 (25, 100) 6 1	78.1 (62.5, 100) 4 3	9.4 (-25, 37.5) 4 3
Bodily pain	12.5 (0, 50) 10 1	20.8 (0, 100) 6 5	31.3 (0, 100) 4 7	25 (-25, 100) 4 7	46.4 (0, 100) 7 0	33.3 (0, 100) 6 1	18.8 (0, 50) 4 3	-6.3 (-50, 25) 4 3
General Health	45.5 (0, 75) 11 0	37.5 (25, 50) 6 5	56.3 (50, 75) 4 7	18.8 (0, 50) 4 7	71.4 (0, 100) 7 0	58.3 (25, 100) 6 1	43.8 (25, 50) 4 3	-31.3 (-50, -25) 4 3
Vitality	54.5 (25, 100) 11 0	58.3 (25, 100) 6 5	68.8 (50, 75) 4 7	18.8 (-25, 50) 4 7	64.3 (50, 100) 7 0	54.2 (25, 100) 6 1	50 (25, 100) 4 3	-6.3 (-25, 25) 4 3
Social Functioning	65.9 (25, 100) 11 0	66.7 (25, 100) 6 5	50 (0, 100) 4 7	0 (-75, 75) 4 7	46.4 (0, 100) 7 0	70.8 (25, 100) 6 1	75 (50, 100) 4 3	25 (0, 50) 4 3
Role Emotional	58 (25, 100) 11 0	68.8 (37.5, 100) 6 5	53.1 (25, 75) 4 7	12.5 (-12.5, 37.5) 4 7	39.3 (0, 75) 7 0	50 (25, 100) 6 1	59.4 (12.5, 87.5) 4 3	21.9 (0, 37.5) 4 3
Mental Health	55.7 (37.5, 75) 11 0	52.1 (37.5, 75) 6 5	53.1 (37.5, 50) 4 7	-12.5 (-25, 12.5) 4 7	41.1 (25, 62.5) 7 0	54.2 (37.5, 62.5) 6 1	43.8 (50, 62.5) 4 3	15.6 (0, 25) 4 3
EQ-5D-3L imaginable health scores	74.5 (40, 100) 11 0	75 (57, 95) 6 5	64.3 (37, 90) 4 7	9.3 (-23, 40) 4 7	65 (20, 80) 7 0	59.2 (30, 85) 6 1	70 (30, 85) 4 3	5 (-5, 10) 4 3
Social Functioning Questionnaire - Global scores	3.6 (2.4, 3.9) 4 7	3.4 (2.3, 3.8) 7 4	3.2 (2.5, 3.8) 4 7	0.1(0.1, 0.1) 1 10	3.3 (2.8, 4) 4 3	3.5 (3.2, 4) 3 4	3.6 (3.3, 3.9) 2 5	0.2(-0.1, 0.5) 2 5
CSRI Total cost of services £	29,744 (945, 91, 547) 11 0	2,914 (286, 7, 575) 6 5	1,799 (682, 3, 718) 4 7	-22,776 (-67, 904, -464) 4 7	1,898 (38, 5, 872) 7 0	2,553 (76, 5, 015) 6 1	1,940 (0, 5, 434) 4 3	-191 (-862, 1, 208) 4 3

BPRS, Brief Psychiatric Rating Scale; WLQ, Work Limitation Questionnaire; CSRI, Client Service Receipt Inventory; EQ-5D-3L, European quality of life scale; SF12-v2, Short Form 12 item health survey; NA, not applicable.

For the IPS group, there was a trend towards reduction in BPRS scores and increases in self-esteem scores. For controls, there was a trend towards reduction in BPRS, self-esteem, and SF12-v2 vitality scores. The IPS group had lower scores than controls on the WLQ at 6 months, indicating lower impact of illness on the ability to work. No clear changes in EQ-5D and SFQ scores were noted. CSRI unit costs reduced over time for the groups, though IPS was more expensive (£29,744 v. £1,898). At baseline, one IPS participant was admitted to a forensic psychiatric hospital (153 days) in the preceding 6 months. This in conjunction with the cost of employing the employment specialist accounts for the higher costs in the IPS group. No further admissions were recorded in the study. One person was recalled to prison owing to breach of license conditions. No further incidents of reoffending were recorded.

## Discussion

### Feasibility Issues

This study sought to examine the feasibility of conducting a full cluster RCT to assess the effectiveness of IPS in improving employment and psychosocial outcomes, as well as reduction in reoffending rates for patients with offending histories. The recruitment rate was 38.3 and the completion rate for IPS was 54.5, with 45.5% acceptability rating. Completion rate for the primary efficacy outcome was near complete. However, completion rates for secondary outcomes for the groups at baseline and 12 months was suboptimal, ranging from 22.2 to 100%. Taken together, the results suggest that it is not feasible to conduct a full RCT in community forensic settings in the UK. Therefore, we did not compute the parameters required to conduct a trial of this kind.

The study faced several challenges which might have caused recruitment and retention difficulties. The study was conducted on a small scale involving a relatively small pool of patients who were on the caseloads of the community forensic services, and due to funding constraints the IPS model was implemented over a short period of time, only 6 months. Additionally, qualitative data involving in-depth interviews with staff, patients, and employers, identified several barriers to IPS implementation in the present study (38, 39). These included competing interests between IPS and psychological therapies, negative attitudes among clinicians about IPS, difficulty engaging employers, lack of employment related performance indicators in health services, and concerns about the impact of returning to work on welfare benefits. Additionally, negative attitudes among clinical staff about patients’ readiness for work were recorded, subjectively determining if the patient was work ready, and holding back referrals. Besides, employers identified offending history, rather than mental health, as a major barrier to employing patients with offending histories. Another important barrier was that National Health Service (NHS) policies prevented the employment specialist and patients from collaborating on job searching and applications together using NHS computers. While the study team tried to minimize these barriers by providing support and information to clinical staff and patients, it is possible that a combination of these factors hampered recruitment and retention, and affected the motivation of patients and mental health professionals to utilise the IPS service.

Research on IPS implementation in forensic mental health settings is an emerging field and previous studies highlighted several barriers to IPS implementation in such settings. In the USA, poor engagement with vocational services, substance use, general medical problems, lack of work skills, and criminal justice system problems were identified as the main barriers to employment in people with severe mental illness and criminal justice involvement (34, 57). In the UK, lack of employment support costs (e.g., criminal record checks, uniforms, travel to interviews) have been identified as additional barrier (30). Furthermore, employers may reluctant to employ people with offending histories especially sex offenders (24).

While the IPS model originated in the USA, several studies demonstrated that IPS can transport successfully to other countries including the UK (58, 59). However, the challenges associated with the implementation of IPS in different social and economic contexts may prevent IPS services from attaining high fidelity (60, 61). These in conjunction with the fact that our study was conducted in a different legal jurisdiction may explain why it was feasible to conduct a study of IPS for patients with offending histories in the USA, but not the UK. Conducting a fully powered trial of IPS for patients with offending histories in community forensic mental health settings in the UK might be feasible in the future if participants were recruited from a larger pool of patients, drawn from multiple sites, and over a longer period of time than the 6 months recruitment period in the present study. Conducting a study of this kind would require an implementation period of at least 12 months to embed the IPS model into clinical services and strategies to address the challenges associated with IPS implementation in community forensic mental health settings. These strategies might include enhancing IPS practices by providing staff training to address negative attitudes about IPS, helping patients manage the stigma attached to offending history, enhancing facilitators to IPS implementation and developing or joining IPS learning collaboratives to foster a culture of collaboration and knowledge sharing between IPS services (62, 63).

### IPS Fidelity

The fidelity reviews showed that IPS implementation was suboptimal in the current study, likely due to the challenges associated with implementation of IPS in community settings in the UK, where IPS is not structurally integrated with psychiatric services (64). Worthy of note here are facilitators to IPS implementation, which included clear communication of the benefits of IPS to stakeholders, support from healthcare managers, and interdisciplinary collaboration. Additionally, we would argue that optimism and an ability to convey it to the jobseeker, the employer and the clinical team is a vital attribute for an employment specialist. Furthermore, development of IPS specifically for individuals with offending histories is an adaptation suggested by some authors (34). Such a model needs to consider the challenges associated with helping these individuals find open employment. Additionally, flexibility and a willingness to consider alternatives to competitive employment, such as volunteering or education, at least initially, may be required for successful implementation of IPS within forensic settings.

### Change in Outcome Measures

The proportions of people in open employment at 12 months were 9.1 and 0% for IPS and controls respectively. However, it must be noted that assessing the effectiveness of IPS was beyond the scope of this study and as such no definitive conclusions can be drawn about the effectiveness of IPS in community forensic mental health settings based on the results of this study. The study in the USA by Bond and colleagues (34) demonstrated that whilst IPS was effective in helping people with severe mental illness and justice involvement enter competitive employment, the study outcomes were less favorable than those achieved in other studies.

### Study Strengths and Weaknesses

This study provides helpful insights into the feasibility of conducting a full RCT into the effectiveness of IPS in improving employment and psychosocial, and reoffending outcomes for patients with offending histories in community forensic mental health settings in the UK, an area that has attracted little attention in the literature. However, the study was conducted on a small scale and failed to recruit the target number owing to recruitment and retention difficulties. Additionally, the study was implemented over a relatively short period of time owing to funding restraints.

## Conclusion

The findings of this study suggest that it is not feasible to conduct a full cluster RCT to assess the effectiveness of IPS in community forensic psychiatric settings in the UK. Conducting a trial of this kind would require a large pool of patients from multiple sites across the UK and a long implementation period (at least 12 months) and recruitment period (at least 18 months), with considerable funding implications, in terms of both research and treatment costs. Further, future studies should address the challenges associated with implementation of IPS in community forensic mental health settings and those related to enabling patients with offending histories to enter competitive employment. Whilst entering competitive employment is a core principle of the IPS model, it is our experience that volunteering and educational opportunities ought to be considered alongside paid work, at least initially, for patients with offending histories due to their lack of recent work experience and work skills. Further, it is also our experience that concerns about stigma might prevent some participants from disclosing vital information about their mental health and offending histories to potential employers, thereby limiting opportunities to provide support to the employers.

## Data Availability Statement

The datasets generated for this study are available on request to the corresponding author.

## Ethics Statement

The studies involving human participants were reviewed and approved by The East Midlands-Nottingham 1 Research Ethics Committee (Ref. 15/EM/0253). The patients/participants provided their written informed consent to participate in this study.

## Author Contributions 

All authors contributed to the design and implementation of study protocol. ET collected the data. SB analyzed the data. NK drafted the manuscript, and all authors reviewed the manuscript.

## Funding 

This article presents independent research funded by the National Institute for Health Research (NIHR) under its Research for Patient Benefit (RfPB) Program (Grant Reference Number PB-PG-1013-32093. The views expressed are those of the authors and not necessarily those of the National Health Service, the NIHR or the Department of Health and Social Care.

## Conflict of Interest

The authors declare that the research was conducted in the absence of any commercial or financial relationships that could be construed as a potential conflict of interest.
